# Effects of Dietary *Lonicera flos* and *Sucutellaria baicalensis* Mixed Extracts Supplementation on Reproductive Performance, Umbilical Cord Blood Parameters, Colostrum Ingredients and Immunoglobulin Contents of Late-Pregnant Sows

**DOI:** 10.3390/ani14142054

**Published:** 2024-07-12

**Authors:** Chengkun Fang, Xiaopeng Tang, Qingtai Zhang, Qifang Yu, Shengting Deng, Shusong Wu, Rejun Fang

**Affiliations:** 1 College of Animal Science, Hunan Agricultural University, Changsha 410128, China; clark_fang@163.com (C.F.); zhangqt00@126.com (Q.Z.); dengshengting97@163.com (S.D.); 2 State Engineering Technology Institute for Karst Desertfication Control, School of Karst Science, Guizhou Normal University, Guiyang 550025, China; tangxiaopeng110@126.com; 3 College of Life Science, Hunan Normal University, Changsha 410081, China; yuqf@hunnu.edu.cn

**Keywords:** colostrum ingredients, immunity, plant extracts, reproductive performance, sows

## Abstract

**Simple Summary:**

Plant extracts (PEs) are a complex mixture of compounds that have been reported to possess multiple biological functions, including antioxidant, immunomodulatory, anti-inflammatory, and anti-microbial activities. PEs have become a viable alternative to antibiotics in animal diets, including sows. The present study showed that dietary *Lonicera flos* and *Sucutellaria baicalensis* mixed extract (LSE) supplementation improved the reproductive performance, immune status, reproductive hormone levels, and colostrum immunoglobulin contents in sows. This study can provide a reference for the rational utilization and application of honeysuckle and *Scutellaria scutellaria* in the sow diet.

**Abstract:**

The present study aimed to determine the effects of dietary *Lonicera flos* and *Sucutellaria baicalensis* mixed extract (LSE) supplementation during the late-pregnancy period on the reproductive performance, umbilical cord blood hematological parameters, umbilical cord serum biochemical parameters, immune indices, hormone levels, colostrum ingredients, and immunoglobulin contents of sows. A total of 40 hybrid pregnant sows were randomly assigned to the control group (CON; sows fed a basal diet) and LSE group (LSE; sows fed a basal diet supplemented with 500 g/t PE). The results indicated that dietary LSE supplementation significantly increased (*p* < 0.05) the number of alive and healthy piglets and the litter weight at birth, and significantly increased (*p* < 0.05) the platelet counts in umbilical cord blood. Dietary LSE supplementation significantly increased (*p* < 0.05) the levels of prolactin (PRL) and growth hormone (GH), and the content of interleukin 2 (IL-2) in umbilical cord serum. Moreover, immunoglobulin A (IgA) and immunoglobulin M (IgM) in the colostrum were increased with PE supplementation (*p* < 0.05). In conclusion, dietary LSE supplementation in late-pregnancy sows could improve reproductive performance and colostrum quality, and could also regulate the levels of reproductive hormone in umbilical cord serum.

## 1. Introduction

In modern swine production, the health status and reproductive performance of sows directly affect productivity and economic benefits [1,2]. However, problems with poor reproductive performance and poor milk quality exist widely in sow production [3,4,5], which has led to an increase in the number of low-birth-weight piglets and a low piglet survival rate. In addition, sows often face metabolic stress during the pregnancy period when attempting to meet the requirements of mammary development and fetal growth [6,7]. Colostrum, produced by mammals immediately after giving birth, is rich in nutrients, immunoglobulins, and growth factors [8]. The living environment of newborn piglets undergoes significant changes after birth, and these changes are combined with the piglets’ low energy reserves and lack of immune protection [9,10]. Therefore, piglets require energy and immune substances from sow colostrum to adapt to the challenging external environment [11]. Improving the quality of sow colostrum is crucial for piglet health. Nutritional regulation during pregnancy is currently an effective strategy to improve sow health status and lactation performance and increase the number of healthy piglets [12,13].

Plant extracts (PEs) are a complex mixture of compounds that contain many active substances such as flavonoids, volatile oils, and organic acids [14,15]. PEs have been reported to possess multiple biological functions, including antioxidant, immunomodulatory, anti-inflammatory, and anti-microbial properties [16,17,18], and have become a viable alternative to antibiotics in animal diets, including those of sows [19,20]. Previous studies have shown that PEs can promote the health and lactation performance of sows [21,22]. For example, sow diets supplemented with silymarin, a plant extract, could alleviate oxidative stress transiently and increase the level of prolactin in late pregnancy [21]. Moreover, dietary seaweed extract supplementation in sows was shown to improve the intestinal health and increase the average daily weight gain [22]. However, there are few studies on the effects of mixed extracts of *Lonicera flos* and *Sucutellaria baicalensis* (LSE) in late-pregnancy sows. In this study, we hypothesized that LSE would have good effects on the reproductive performance, immune status, and colostrum quality of sows. Therefore, the objective of this study was to investigate the effect of dietary supplementation with LSE on the reproductive performance, hematological parameters in umbilical cord blood, biochemical parameters, immune indices and hormone levels in umbilical cord serum, and ingredient and immunoglobulin contents in the colostrum of sows during late pregnancy, thereby providing a reference for the rational utilization and application of *Lonicera flos* and *Sucutellaria baicalensis* in the sow diet.

## 2. Materials and Methods

### 2.1. Source of Plant Extract

LSE was provided by CENTRE (Inner Mongolia) Technology Co., Ltd. (Xilingol, China). The composition of LSE was the mixed extracts of honeysuckle and *Sucutellaria baicalensis*, and the effective ingredients were chlorogenic acid and baicalin, among which the chlorogenic acid content was 0.22% and the baicalin content was 2.20%.

### 2.2. Sows, Diet, and Experimental Design

A total of 40 hybrid pregnant sows (Jiahe Agriculture and Animal Husbandry Co., Ltd., Changsha, China) with similar parity (second parity) and pregnancy (85 days) were randomly allocated into the control group (CON; sows fed a basal diet) and the LSE group (LSE; sows fed a basal diet supplemented with 500 g/t PE) with 20 replicates each. The basal diet (Table 1) was formulated to meet or exceed the requirements of pregnant sows as outlined by the National Research Council (NRC, 2012).

Prior to the commencement of the experiment, the housing facility for the sows was thoroughly cleaned and disinfected. The pregnant sows were placed in a crate with half-slatted floors. Then, a 3-day pre-trial was conducted, during which the late-pregnancy diet was adapted. The sows were given daily chow rations weighing 3% of their body weight. The sows were fed twice a day at 7:00 and 15:00 with ad libitum access to water. Seven days before birth, the sows were moved into a farrowing building in farrowing pens.

### 2.3. Performance of Sows

Feed intake was recorded daily. The reproductive performance parameters employed the methods described by Wang et al. [23]. At parturition, the number of alive, healthy, weak, mummies, and stillborn piglets were recorded. Then, the body weight of the newborn piglets was recorded.

The rate of healthy and stillborn piglets was calculated by the following formula: healthy birth rate (%) = number of healthy piglets/number of total piglets × 100; stillborn rate (%) = number of stillborn piglets/number of total piglets × 100.

### 2.4. Sample Collection

At parturition, blood samples of approximately 10 mL were collected from the umbilical cord. Each sample was divided into 2 subsamples. One subsample was used for hematological analysis within 4 h of collection, while the other subsample was centrifuged at 3500× *g* for 10 min at 4 °C and stored at −20 °C for biochemical indices measurement.

After the birth of the last piglet, two portions of colostrum (10 mL each) were taken from each sow and stored at −20 °C for analysis.

### 2.5. Umbilical Cord Blood Parameters

The umbilical cord blood quality parameters, including white blood cell (WBC), red blood cell (RBC), red cell distribution width—coefficient of variation (RDW-CV), hematocrit (HCT), hemoglobin concentration (HGB), mean corpuscular hemoglobin (MCH), mean corpuscular volume (MCV), mean cell hemoglobin concentration (MCHC), red cell distribution width—standard deviation (RDW-SD), mean platelet volume (MPV), platelet distribution width (PDW), platelet count (PLT), lymphocyte percentage, neutrophil percentage, and monocyte percentage were measured using a hematology blood analyzer (BC-600, Shenzhen Mindray Bio-Medical Electronics Co., Ltd., Shenzhen, China) according to the manufacturer’s instructions.

The umbilical cord serum concentration of total protein (TP), albumin (ALB), urea, glucose (GLU), total cholesterol (TC), and total glyceride (TG) was examined using an automated biochemical analyzer (Mindray BS-420, Shenzhen Mindray Bio-Medical Electronics Co., Ltd., Shenzhen, China).

The contents of interleukin (IL)-2, IL-6, tumor necrosis factor-α (TNF-α), estrogen (E), prolactin (PRL), and growth hormone (GH) were determined using enzyme-linked immunosorbent assay (ELISA) kits, following the protocol provided by the manufacturer (Shenzhen Mindray Bio-Medical Electronics Co., Ltd., Shenzhen, China).

### 2.6. Colostrum Ingredients

The ingredients of the colostrum were evaluated for various parameters such as protein percentage, fat percentage, lactose percentage, urea nitrogen, content, non-milk fat solid content, and total dry matter using a fully automated milk analyzer (MilkoScan ^TM^ FT+200, FOSS, Hilleroed, Denmark). Concurrently, the somatic cell count was determined with a cell analyzer (Type 79910, Fossomatic FC, FOSS, Hilleroed, Denmark), strictly adhering to the manufacturer’s instructions.

### 2.7. Colostrum Immunoglobulins Content

The contents of immunoglobulin M (IgM), immunoglobulin A (IgA), and immunoglobulin G (IgG) in the colostrum were measured with enzyme immunoassay kits (Shenzhen Mindray Bio-Medical Electronics Co., Ltd., Shenzhen, China) in accordance with the protocol of the manufacturer.

### 2.8. Statistical Analysis

All data were subjected to an unpaired t-test using SPSS 26.0 programs (SPSS, Inc., Chicago, IL, USA). The results were expressed as means ± standard deviation (SD), and differences were considered significant at *p* < 0.05.

## 3. Results

### 3.1. Reproductive Performance of Sows

The reproductive performance of the sows is presented in Table 2. The pregnant sows fed diets with LSE had an increased number of live piglets and healthy piglets, and greater litter weight at birth (*p* < 0.05), and there was a trend of increased feed intake (*p* = 0.07). The dietary addition of LSE had no effect on the average litter size, the number of weak piglets, the number of stillborn piglets, the number of mummified piglets, the healthy birth rate, the stillbirth rate, or the average litter weight at birth.

### 3.2. Umbilical Cord Blood Hematological Parameters

As shown in Table 3, the pregnant sows fed diets with added LSE exhibited an increased platelet count (*p* < 0.05) and increased hemoglobin concentration (*p* = 0.080). However, no significant differences were observed in terms of the white blood cell count, red blood cell count, hematocrit ratio, mean corpuscular volume, mean erythrocyte hemoglobin content, mean erythrocyte hemoglobin concentration, coefficient of variation of erythrocyte distribution width, standard deviation of red blood cell distribution width, mean platelet volume, platelet distribution width, percentage of neutrophils, percentage of lymphocytes, or percentage of monocytes.

### 3.3. Umbilical Cord Serum Biochemical Parameters

Table 4 shows that no significant differences were observed in the contents of ALB, urea, TG, TC, TP, or Glu between the CON and LSE groups. However, the pregnant sows fed diets with added LSE showed a tendency for increased TG (*p* = 0.094) and TC (*p* = 0.062) content.

### 3.4. Umbilical Cord Serum Immune Indices

As shown in Table 5, the pregnant sows fed the diet with added PE exhibited increased IL-2 content (*p* < 0.05). However, the dietary addition of LSE had no effect on the contents of IL-6 or TNF-α.

### 3.5. Umbilical Cord Serum Hormone Levels

As shown in Table 6, the dietary supplementation of LSE increased the contents of PRL and GH in the sows fed this diet compared to those fed the CON diet (*p* < 0.05). However, there was no significant difference in the content of E between the CON and PE groups.

### 3.6. Colostrum Ingredients

As shown in Table 7, no significant differences were observed for the somatic cell count, colostrum protein percentage, colostrum fat percentage, colostrum lactose percentage, urea nitrogen content, non-milk-fat solid content, and total dry matter between the CON and LSE groups.

### 3.7. Colostrum Immunoglobulin Content

Table 8 shows that LSE supplementation significantly increased the contents of IgM and IgA in the colostrum (*p* < 0.05). However, there was no significant difference in the content of IgG between the LSE group and the CON group.

## 4. Discussion

The reproductive capacity of sows plays a key role in determining the efficiency of pig production [24]. Sows may experience significant stress during late pregnancy, leading to decreased reproductive performance [25,26]. Therefore, it is crucial to find suitable feed additives to improve the reproductive performance of sows. Previous studies have confirmed that dietary PE supplementation is very helpful to improve the reproductive performance of sows [27,28,29]. Among them, *Lonicera flos* [30] and *Scutellaria baicalensis* [31] are two widely used traditional Chinese herbal medicines, and their extracts have good antioxidant, antibacterial, anti-inflammatory, and immunomodulatory effects. However, there are few reports about the effects of mixed extracts of *Lonicera flos* and *Scutellaria baicalensis* on the reproductive performance of pregnant sows. In the present study, dietary LSE administration increased the number of live piglets and healthy piglets and the litter weight at birth, and reduced the stillbirth rate and the number of mummified piglets. These findings indicated that dietary LSE supplementation significantly improved the reproductive performance of these sows. In accordance with our study, Wang et al. [32] reported that the supplementation of 1.0 g/kg *Scutellaria baicalensis* and *Lonicera japonica* mixed extracts in the diets of late-pregnant sows increased the number of live piglets, litter birth weight, and average daily feed intake of sows. Lonicera japonica and *Lonicera flos* are very similar, with chlorogenic acid being one of their main active components. These results suggested that supplementation of pregnant sow diets with plant extracts could improve reproductive performance.

It is well known that blood parameters can be used to reflect the health status of animals [33]. The placenta and umbilical cord play major roles in the transfer of nutrition, gas, and metabolites between mother and developing fetus during pregnancy [34,35]. Therefore, the monitoring of umbilical cord blood parameters is important for both sows and fetuses. Usually, blood hematological parameters mainly include the correlation analysis of red blood cells, white blood cells, and platelets [36]. In this study, the hematological parameters in the umbilical cord blood were within the reference value ranges established for pregnant sows. Platelets are involved in hemostasis, inflammation, and bacteriostatic activity [37]. In our study, the dietary LSE supplementation increased the platelet count in the umbilical cord blood. The umbilical cord is the bridge between the sow and the piglets. The increased platelet counts observed in the umbilical cord blood meant that the dietary supplementation of LSE had improved the immune function of the sows, indicating that one of the mechanisms of LSE may be to improve the reproductive performance of sows, that is, to increase the number of live and healthy piglets by reducing the inflammatory response of the sows.

Previous research confirmed that antibodies can be transported from mother to fetus by the placenta [38,39]. Inflammation and its counteractive processes are significantly mediated by cytokines [39,40]. In the present study, dietary LSE supplementation increased IL-2 levels in the umbilical cord serum of sows. IL-2, an immunomodulatory peptide generated by stimulated T lymphocytes, plays a crucial role in stimulating T-cell growth, engaging in T-cell apoptosis, and demonstrating immunosuppressive properties [41,42]. Although the effect of the dietary addition of LSE on the IL-2 content in the umbilical cord of sows has not been reported, the result of increased IL-2 levels in the umbilical cord of sows fed with LSE in this study suggests that another possible mechanism by which LSE improves the reproductive performance of sows is to increase the number of live and healthy piglets by increasing anti-inflammatory factors.

Pregnancy in sows is a sophisticated biological process that is regulated by the hypothalamic–pituitary–gonadal axis [43]. In addition, physiological changes in the sow can be affected by reproductive hormones during pregnancy [44]. Prolactin (PRL) plays a role in promoting mammary gland growth, increasing lactation performance, and maintaining pregnancy [45]. In our study, dietary PE supplementation increased the level of PRL in the umbilical cord serum. This suggests that the addition of LSE to the diet promoted mammary gland development and improved lactation performance in these sows, which is critical for improving the health of suckling piglets, as breast milk is an important nutrient source for piglets during the lactation stage. Consistent with our findings, Wu et al. [46] demonstrated that dietary supplementation with a mixture of soybean isoflavone and astragalus polysaccharide increased the level of PRL in the serum of lactating sows. Growth hormone is a protein hormone secreted by the pituitary gland [47], playing a role in promoting tissue growth and enhancing body anabolism [48]. In this study, the dietary LSE supplementation increased the level of GH in the umbilical cord serum, which indicated that one of the possible mechanisms of LSE to promote the reproductive performance of sows is to promote fetal growth by increasing the secretion of GH, thereby increasing the number of live and healthy piglets and decreasing the number of weak and mummified piglets. Thus, dietary LSE supplementation could regulate the immune state and reproductive hormone levels in sows.

Colostrum is rich in various nutrients, immunoglobulins, and growth factors, which can provide adequate nutrition and immune protection for newborn piglets, reducing the occurrence of disease and deaths [8]. The nutrient intake of pregnant sows affects colostrum quality [49,50]. Sun et al. [51] reported that the supplementation of 8% Moringa oleifera in late-pregnancy sow diets increased the colostrum protein content, indicating that plant extracts could improve the quality of colostrum in sows. Similarly, in this study, the dietary LSE supplementation increased the contents of fat and protein in the colostrum, although the difference was not significant. Due to newborn piglets’ immature immune system, colostrum intake is the main way for them to obtain immunity. For example, newborn piglets can establish passive immunity by the ingestion of immunoglobulins in colostrum [52]. In our study, the dietary LSE supplementation increased the colostrum IgA and IgM contents. Consistent with our findings, Wang et al. [32] indicated that the supplementation of 1.0 g/kg *Scutellaria baicalensis* and *Lonicera japonica* mixed extracts in the diets of late-pregnant sows increased colostrum IgG and IgA concentrations. According to previous studies, LSE supplementation in late-pregnancy sow diets improved the quality of colostrum and the contents of immunoglobulins. Meanwhile, these results also suggest that the supplementation of plant extracts in the diet of sows in late pregnancy could improve the live birth rate and health of piglets, which may be related to the increase in the IgA and IgM content in the colostrum induced by LSE, thus improving the immune function of piglets after birth.

## 5. Conclusions

In summary, the present study indicated that the dietary LSE supplementation significantly improved the reproductive performance of the sows, as evidenced by the increased number of live and healthy piglets and the greater litter weight at birth. The dietary LSE supplementation also significantly improved the immune status of the sows, as evidenced by the increased platelet counts in the umbilical cord blood and the content of IL-2 in the umbilical cord serum. The dietary LSE supplementation significantly increased the levels of growth hormone prolactin PRL and GH in the umbilical cord serum of the sows. Moreover, the dietary LSE supplementation significantly increased the immunoglobulin A (IgA) and immunoglobulin M (IgM) in the colostrum of the sows. Thus, LSE could improve the reproductive performance and colostrum quality of sows, and could also regulate the levels of reproductive hormones in their umbilical cord serum.

## Figures and Tables

**Table 1:** Composition and nutrient level of basal diet.

Ingredients	Content, %	Item	Nutrient Level
Xingnongtai 950 ^1^	15.00	CP (%)	15.39
Hulled barley	20.00	ME (Kcal/kg)	3205.06
Soybean meal	15.00	Ca (%)	0.93
Corn	10.70	P (%)	0.67
Brown rice	10.00	Lysine (%)	0.95
Beet meal	6.00	Methionine (%)	0.37
Rice bran meal	7.00	Cystine (%)	0.26
Yeast culture	3.50	Threonine (%)	0.68
Expanded soybean	3.00	Tryptophan (%)	0.19
Calcium hydrogen	1.40		
Soybean oil	0.50		
Stone powder	1.00		
Expanded flaxseed	2.00		
Qiaozi ^2^	2.00		
Fungicide	0.20		
Sodium bicarbonate	0.20		
Premix ^3^	2.50		
Total	100.00		

^1^ Xingnongtai 950: 95% imported corn and 5% soybean meal. ^2^ Qiaozi: 50% near-expired Dove chocolate powder and 50% flour. ^3^ The premix provided the following per kilogram: Vitamin A 7000 IU, Vitamin D3 1800 IU, Vitamin E 160 IU, Vitamin B1 2 mg, Vitamin B2 6 mg, Vitamin B6 3 mg, D-pantothenic acid 16 mg, folic acid 6.8 mg, niacin 70 mg, Cu as copper sulfate 12.5 mg, Fe as polysaccharide iron 100 mg, Mn as manganese sulfate 64 mg, and salt 5.4 g. CP, crude protein; P, phosphorus; Ca, calcium; ME, metabolizable energy.

**Table 2:** Effects of dietary LSE supplementation on feed intake and reproductive performance of late-pregnant sows.

Item ^1^	CON	LSE	*p*-Value
Feed intake (kg/sow)	2.96 ± 0.09	2.91 ± 0.08	0.070
Number of average litter size	12.88 ± 1.20	13.80 ± 1.97	0.123
Number of live piglets	10.90 ± 2.61	13.65 ± 3.10	0.004
Number of healthy piglets	10.55 ± 2.42	13.25 ± 3.26	0.005
Number of weak piglets	0.35 ± 0.67	0.40 ± 0.82	0.834
Number of stillborn piglets	0.75 ± 1.02	0.80 ± 1.06	0.880
Number of mummified piglets	0.1 ± 0.31	0	0.163
Healthy birth rate (%)	97.27 ± 5.21	96.78 ± 6.42	0.795
Stillbirth rate (%)	6.62 ± 8.89	5.34 ± 7.12	0.618
Litter weight at birth (kg)	16.88 ± 4.81	19.85 ± 3.98	0.043
Average litter weight at birth (kg)	1.53 ± 0.20	1.42 ± 0.21	0.102

^1^ CON, basal diet; LSE, basal diet + 500 g/t LSE. Results are presented as means ± SD.

**Table 3:** Effects of dietary LSE supplementation on umbilical cord blood hematological parameters from late-pregnant sows.

Item ^1^	CON	LSE	*p*-Value
White blood cells (10^9^/L)	2.69 ± 0.54	2.51 ± 0.68	0.632
Red blood cells (10^12^/L)	5.04 ± 0.60	5.26 ± 0.36	0.501
Hemoglobin concentration (g/L)	87.60 ± 8.08	97.20 ± 7.01	0.080
Hematocrit (%)	30.25 ± 1.82	32.74 ± 2.14	0.107
Mean corpuscular volume (fL)	64.00 ± 2.79	61.98 ± 1.43	0.146
Mean corpuscular hemoglobin (pg)	19.16 ± 0.74	18.72 ± 0.79	0.365
Mean cell hemoglobin concentration (g/L)	304.60 ± 10.38	302.17 ± 16.51	0.782
Red cell distribution width—coefficient of variation (%)	20.43 ± 1.97	19.84 ± 1.41	0.587
Red cell distribution width—standard deviation (fL)	50.12 ± 5.40	50.27 ± 8.16	0.971
Mean platelet volume (fL)	7.02 ± 1.29	6.50 ± 1.38	0.581
Platelet distribution width (%)	17.28 ± 0.47	17.15 ± 0.58	0.671
Neutrophil percentage (%)	29.68 ± 17.64	28.15 ± 12.96	0.867
Lymphocyte percentage (%)	66.57 ± 18.99	65.57 ± 15.45	0.922
Monocyte percentage (%)	3.55 ± 1.75	5.93 ± 3.09	0.131
Platelet count (10^9^/L)	52.77 ± 1.57	82.33 ± 25.72	0.037

^1^ CON, basal diet; LSE, basal diet + 500 g/t LSE. Results are presented as means ± SD.

**Table 4:** Effects of dietary LSE supplementation on umbilical cord serum biochemical parameters of late-pregnant sows.

Item ^1^	CON	LSE	*p*-Value
ALB (g/L)	8.00 ± 1.08	7.04 ± 1.62	0.251
Urea (mmol/L)	5.96 ± 1.57	5.89 ± 2.11	0.953
TG (mmol/L)	0.15 ± 0.02	0.19 ± 0.05	0.094
TC (mmol/L)	1.30 ± 0.11	1.58 ± 0.31	0.062
TP (g/L)	25.15 ± 2.16	23.66 ± 3.47	0.392
Glu (mmol/L)	1.03 ± 0.22	1.34 ± 0.85	0.435

^1^ CON, basal diet; LSE, basal diet + 500 g/t LSE. Results are presented as means ± SD. ALB: albumin; TG: total glyceride; TC: total cholesterol; TP: total protein; Glu: total cholesterol.

**Table 5:** Effects of dietary LSE supplementation on umbilical cord serum immune indices in late-pregnant sows.

Item ^1^	CON	LSE	*p*-Value
IL-2 (pg/mL)	220.00 ± 35.38	268.86 ± 11.24	0.018
IL-6 (pg/mL)	598.30 ± 88.22	601.36 ± 97.21	0.956
TNF-α (pg/mL)	114.33 ± 25.45	116.19 ± 29.00	0.909

^1^ CON, basal diet; LSE, basal diet + 500 g/t LSE. Results are presented as means ± SD. IL-2, interleukin-2; IL-6, interleukin-6; TNF-α, tumor necrosis factor α.

**Table 6:** Effects of dietary LSE supplementation on umbilical cord serum hormone levels in late-pregnant sows.

Item ^1^	CON	LSE	*p*-Value
E (pg/mL)	212.01 ± 36.93	216.97 ± 44.88	0.839
PRL (mIU/L)	510.60 ± 79.63	680.54 ± 95.26	0.007
GH (ng/mL)	15.14 ± 1.61	21.07 ± 1.02	<0.001

^1^ CON, basal diet; LSE, basal diet + 500 g/t LSE. Results are presented as means ± SD. E, estrogen; PRL, prolactin; GH, growth hormone.

**Table 7:** Effects of dietary LSE supplementation on colostrum ingredients in late-pregnant sows.

Item ^1^	CON	LSE	*p*-Value
Somatic cell count (×10^4^ pieces/mL)	739.62 ± 420.94	384.18 ± 157.16	0.136
Protein percentage (%)	18.51 ± 2.63	19.69 ± 3.53	0.527
Fat percentage (%)	3.92 ± 1.35	5.20 ± 1.51	0.175
Lactose percentage (%)	2.21 ± 0.78	2.27 ± 0.30	0.872
Urea nitrogen content (mg/dL)	61.40 ± 20.96	60.60 ± 16.91	0.943
Non-milk-fat solid content (%)	24.41 ± 2.25	25.48 ± 3.04	0.504
Total dry matter (%)	9.60 ± 0.93	10.45 ± 1.12	0.206

^1^ CON, basal diet; LSE, basal diet + 500 g/t LSE. Results are presented as means ± SD.

**Table 8:** Effects of dietary LSE supplementation on colostrum immune function in late-pregnant sows.

Item ^1^	CON	LSE	*p*-Value
IgM (g/L)	11.99 ± 2.12	17.31 ± 1.37	<0.010
IgA (mg/L)	522.60 ± 103.66	740.17 ± 49.23	0.020
IgG (g/L)	11.24 ± 2.00	13.03 ± 2.67	0.217

^1^ CON, basal diet; LSE, basal diet + 500 g/t LSE. Results are presented as means ± SD. IgM, immunoglobulin M; IgA, immunoglobulin A; IgG, immunoglobulin G.

## Data Availability

The original contributions presented in the study are included in the article; further inquiries can be directed to the corresponding authors.
